# Supplementary material for: Morphological and anatomical variations in subcortical anatomy between humans and chimpanzees associated with heritability patterns related to human behavioral traits
Source: Commun Biol. 2026 Apr 21;9:852. doi: 10.1038/s42003-026-10066-6 (PMC13284390; doi:10.1038/s42003-026-10066-6)
Supplement: Supplementary file 1 — Supplementary Information [file 42003_2026_10066_MOESM1_ESM.pdf]

Supplementary Information (SI)

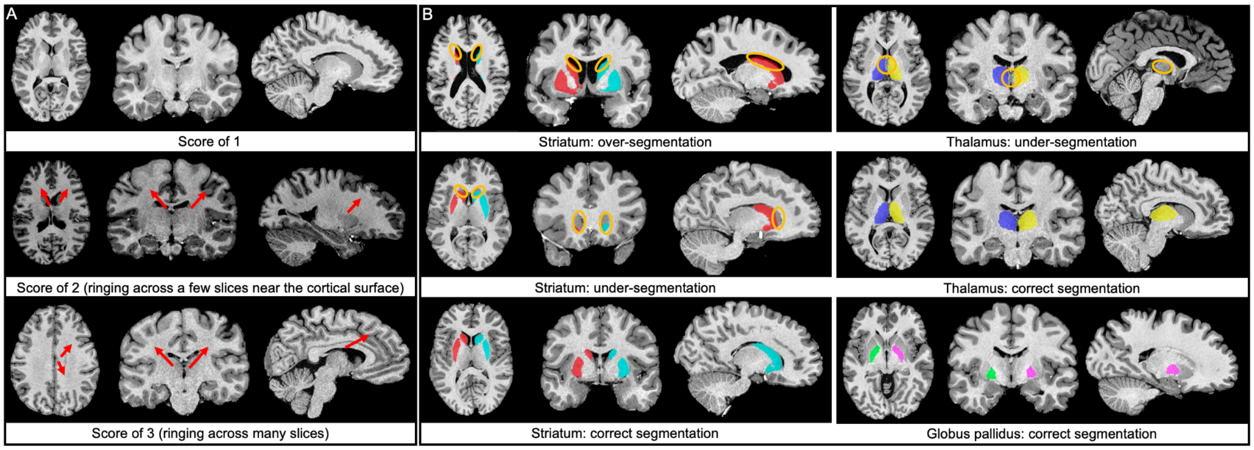

Figure S1. Examples of motion quality control ratings and subcortical structure segmentations. A) Motion quality control ratings after data preprocessing with minc-bpipe; red arrows point in the direction of motion artifacts (ringing); images in each panel are being displayed along axial (left), coronal (center) and sagittal (right) planes. B) Good and bad segmentations for the striatum, thalamus and globus pallidus; orange circles indicate areas that are over or under segmented; images are being displayed along axial (left), coronal (center) and sagittal (right) planes.

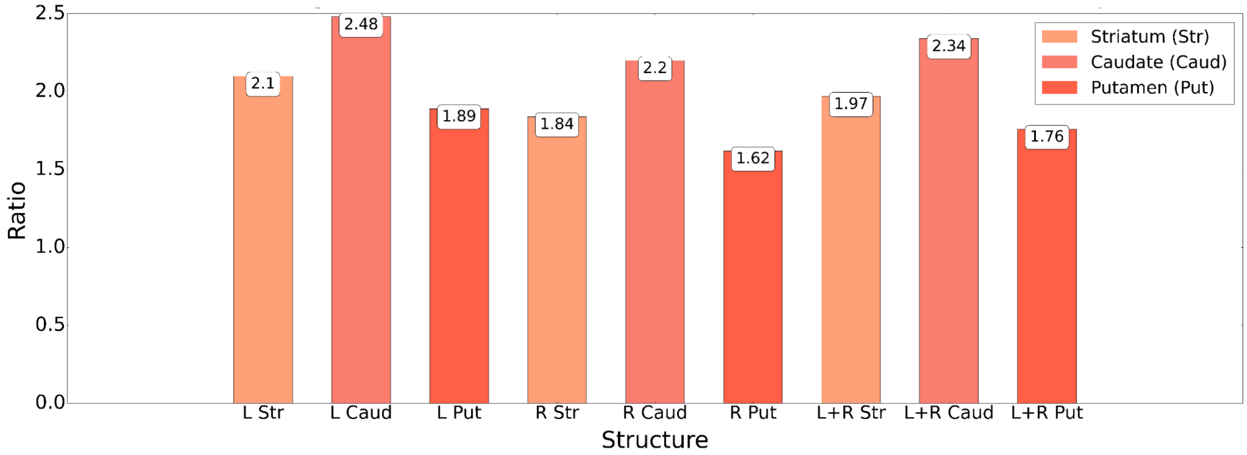

Figure S2. Ratio of striatum-specific volumes in humans relative to striatum-specific volumes in chimpanzees.
Show for the left (L), right (R) and bilateral (L+R) striatum (Str), caudate (Caud) and putamen (Put).

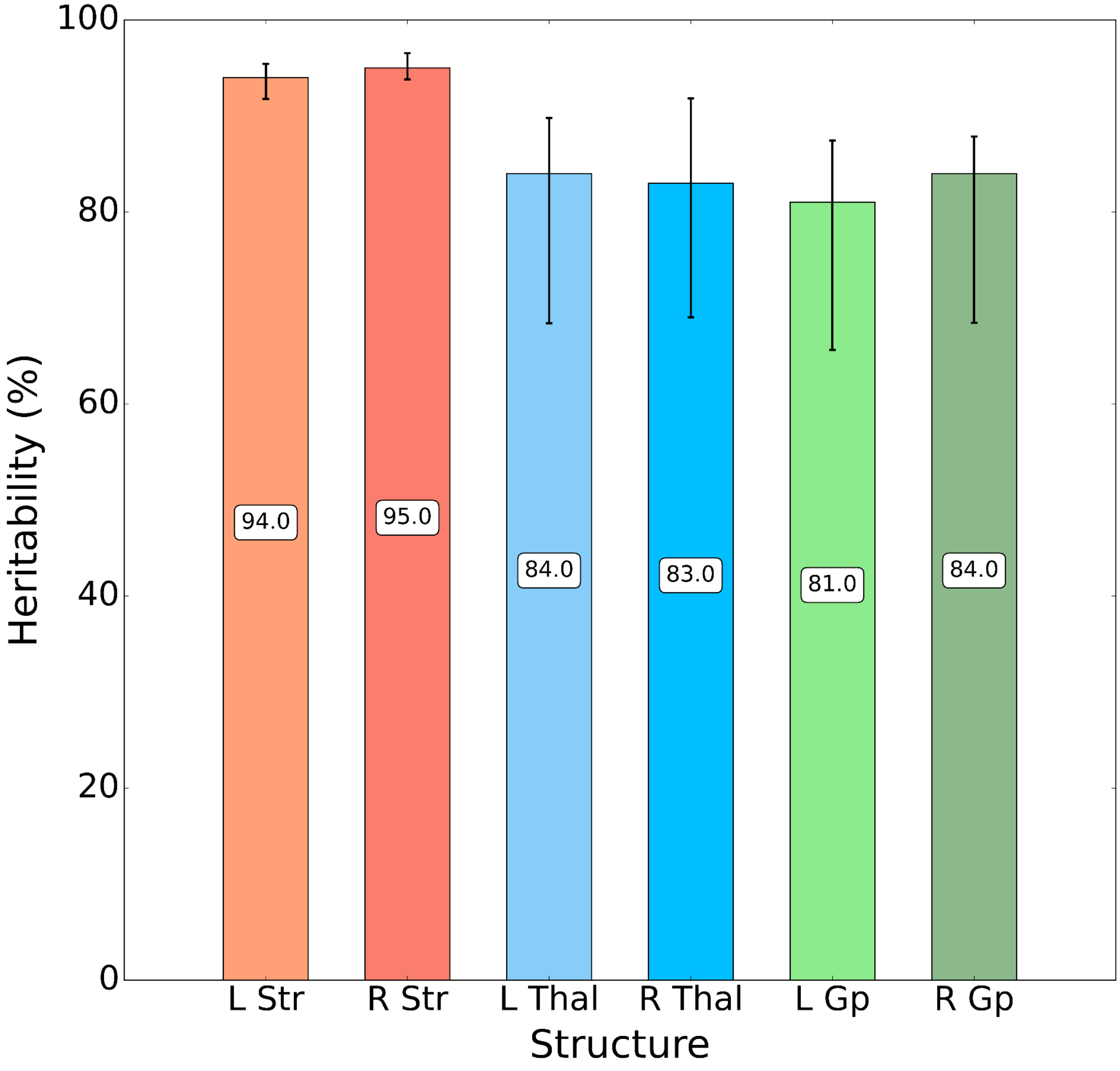

Figure S3. Heritability of ipsilateral structure-specific volumes, adjusted for sex, age and TBV. Shown for the left (L) and right (R) striatum (Str), thalamus (Thal) and globus pallidus (GP). Black bars indicate 95% confidence intervals. Black bars indicate 95% confidence intervals.

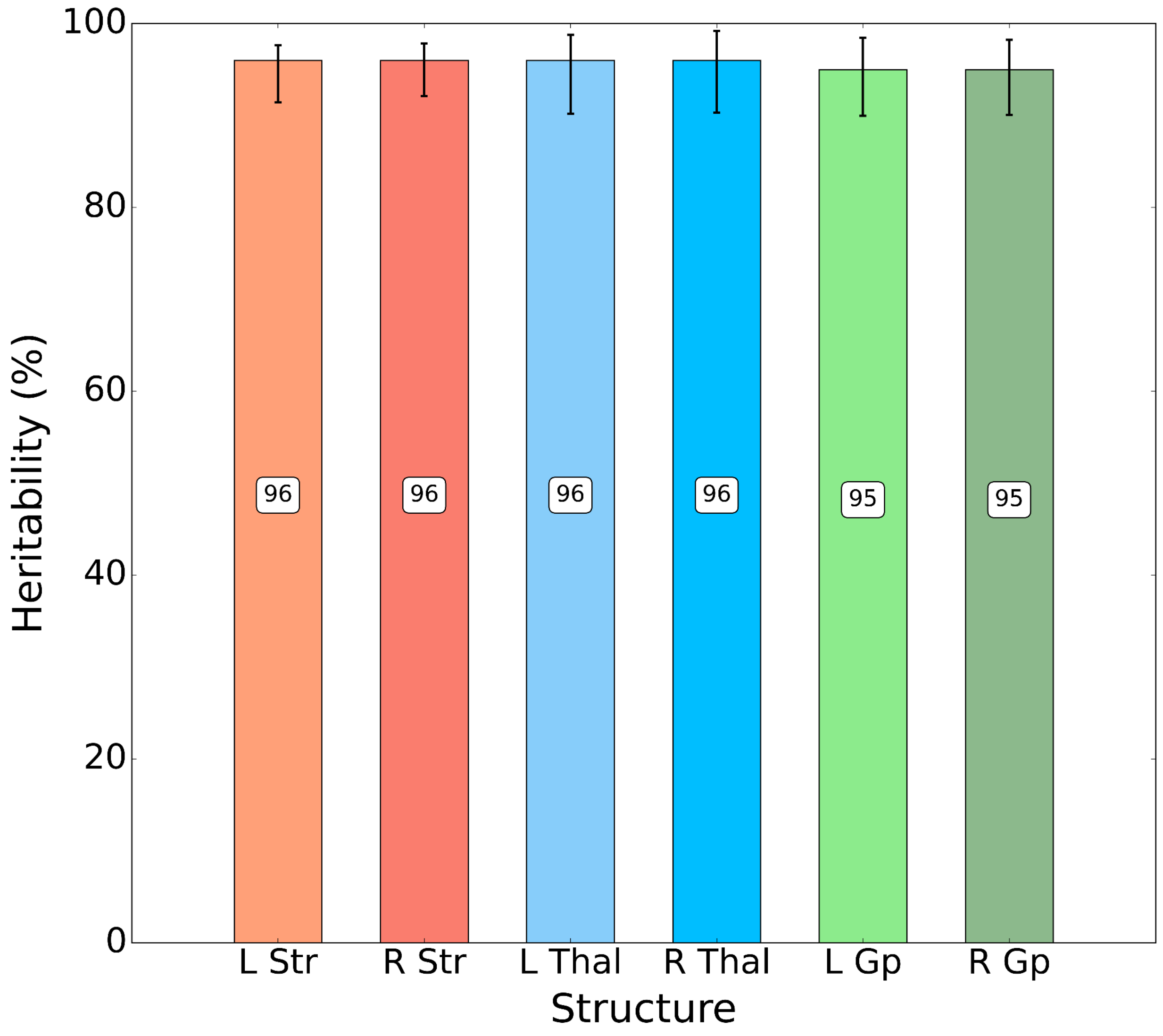

Figure S4. Shared heritability between TBV and ipsilateral structure-specific volumes, adjusted for sex and age.

Shown for the left (L) and right (R) striatum (Str), thalamus (Thal) and globus pallidus (GP). Black bars indicate 95% confidence intervals.

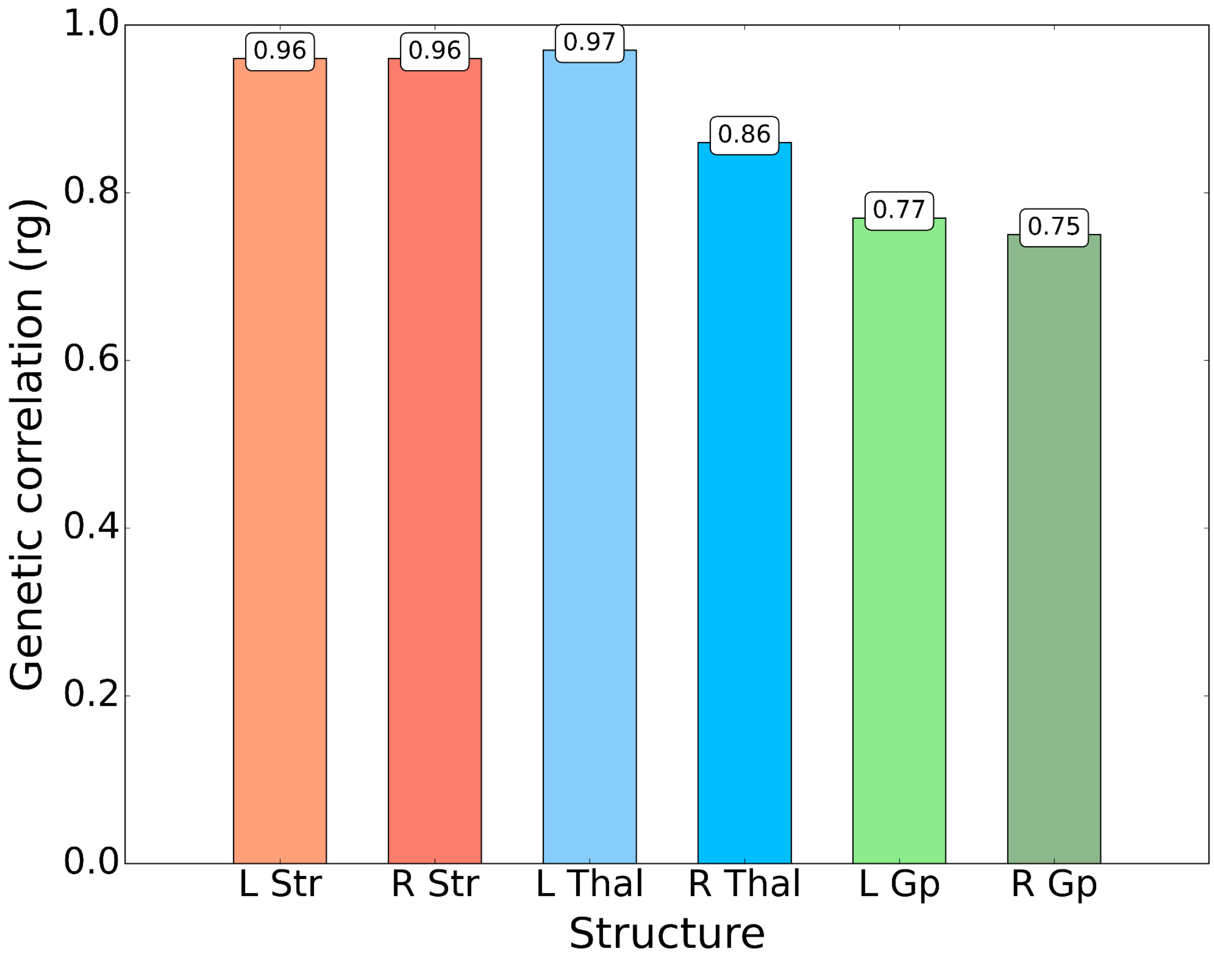

Figure S5. Genetic correlations (rg) between TBV and ipsilateral structure-specific volumes, adjusted for sex and age.

Shown for the left (L) and right (R) striatum (Str), thalamus (Thal) and globus pallidus (GP).

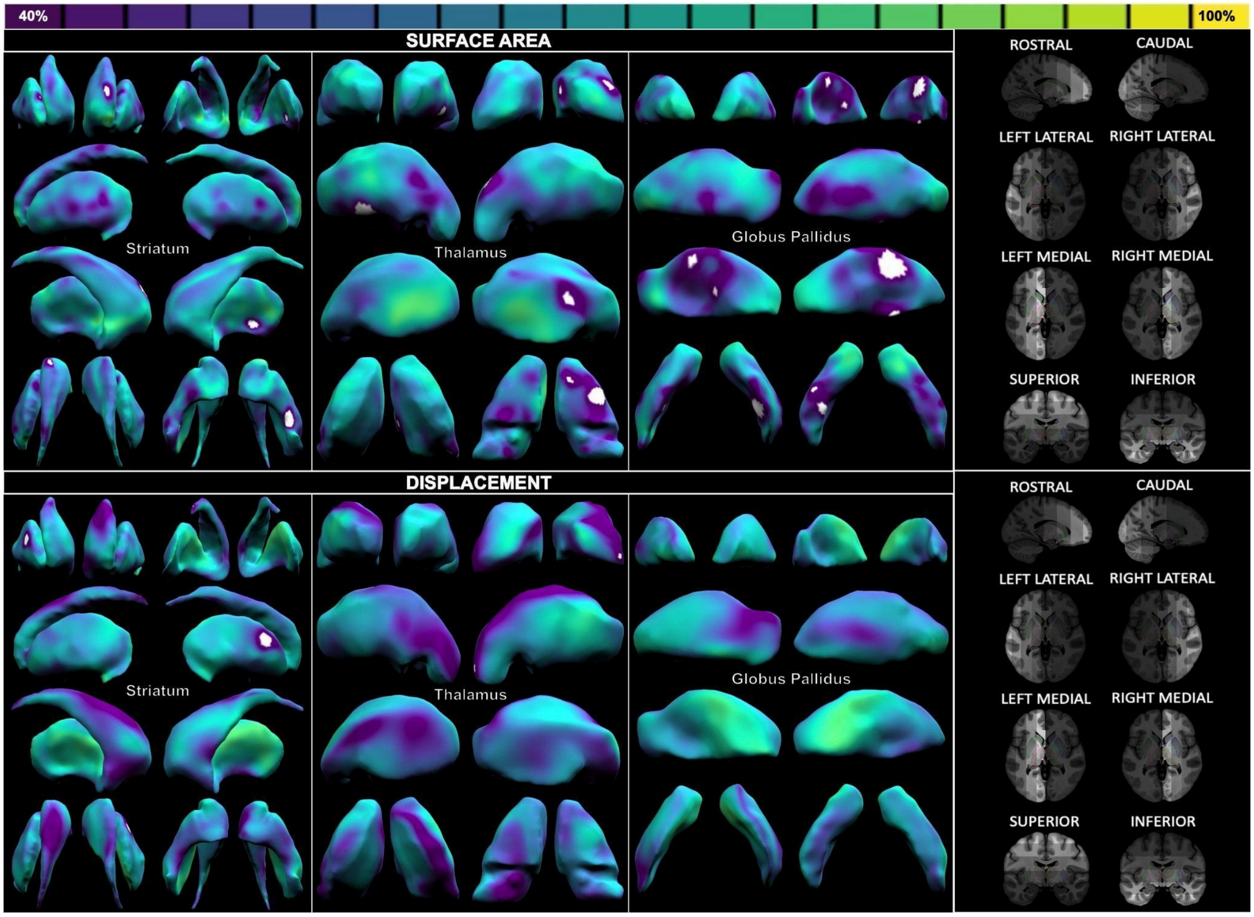

Figure S6. Heritability of sex, age and TBV-adjusted vertex-wise surface area (top row) and displacement (bottom row) in the striatum, thalamus and globus pallidus.
The views of the structures on display are shown in the column on the right-hand side; brighter areas on the brain indicate the view from which the structure is being seen. Values in white were not significant (5% FDR correction). Ranges go from 40% (dark purple) to 100% (yellow).

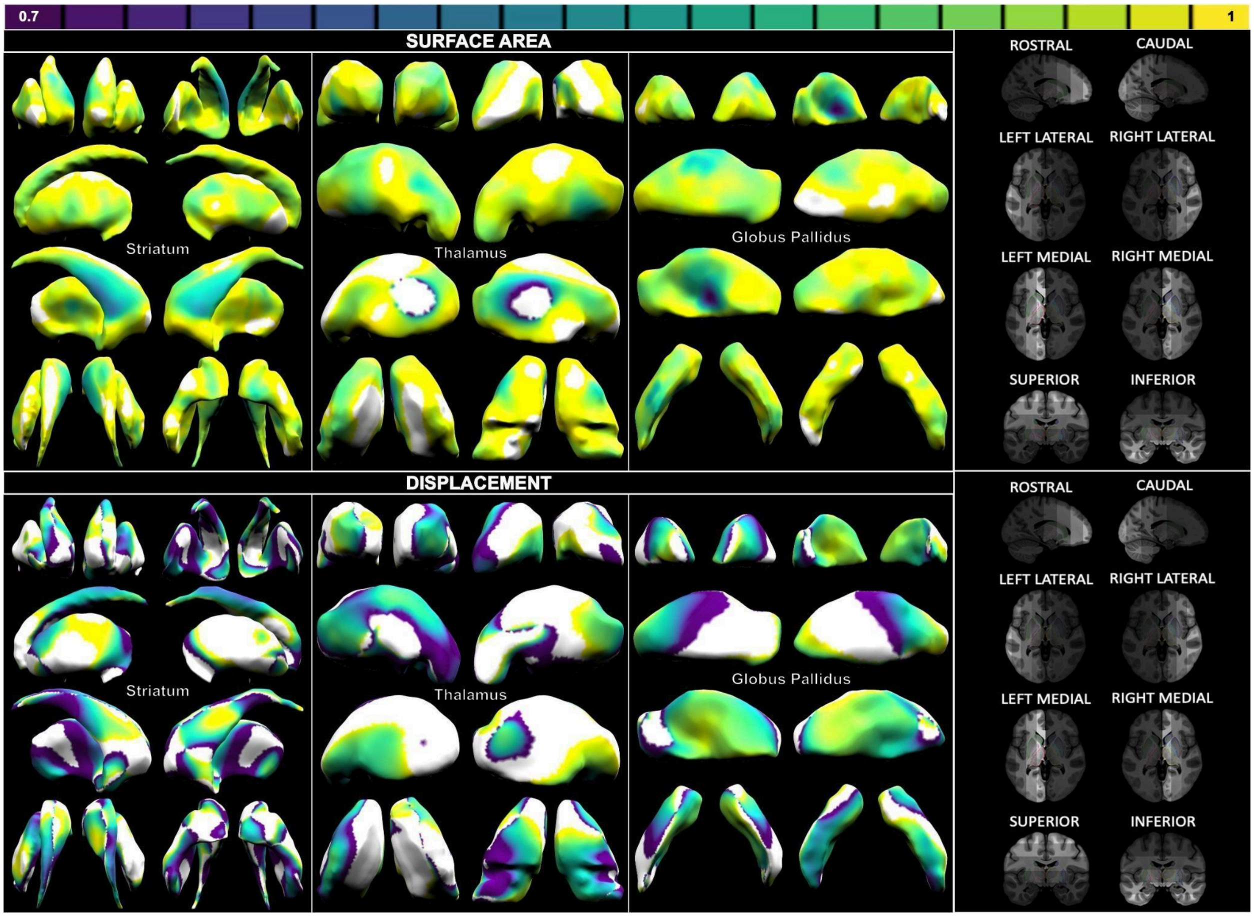

Figure S7. Shared heritability between TBV and sex and age-adjusted vertex-wise surface area (top row) and displacement (bottom row) in the striatum, thalamus and globus pallidus.
The views of the structures on display are shown in the column on the right-hand side; brighter areas on the brain indicate the view from which the structure is being seen. Values in white either failed optimization (heritability value below 0% or above 100%) or 5% FDR correction. Ranges go from 70% (dark purple) to 100% (yellow).

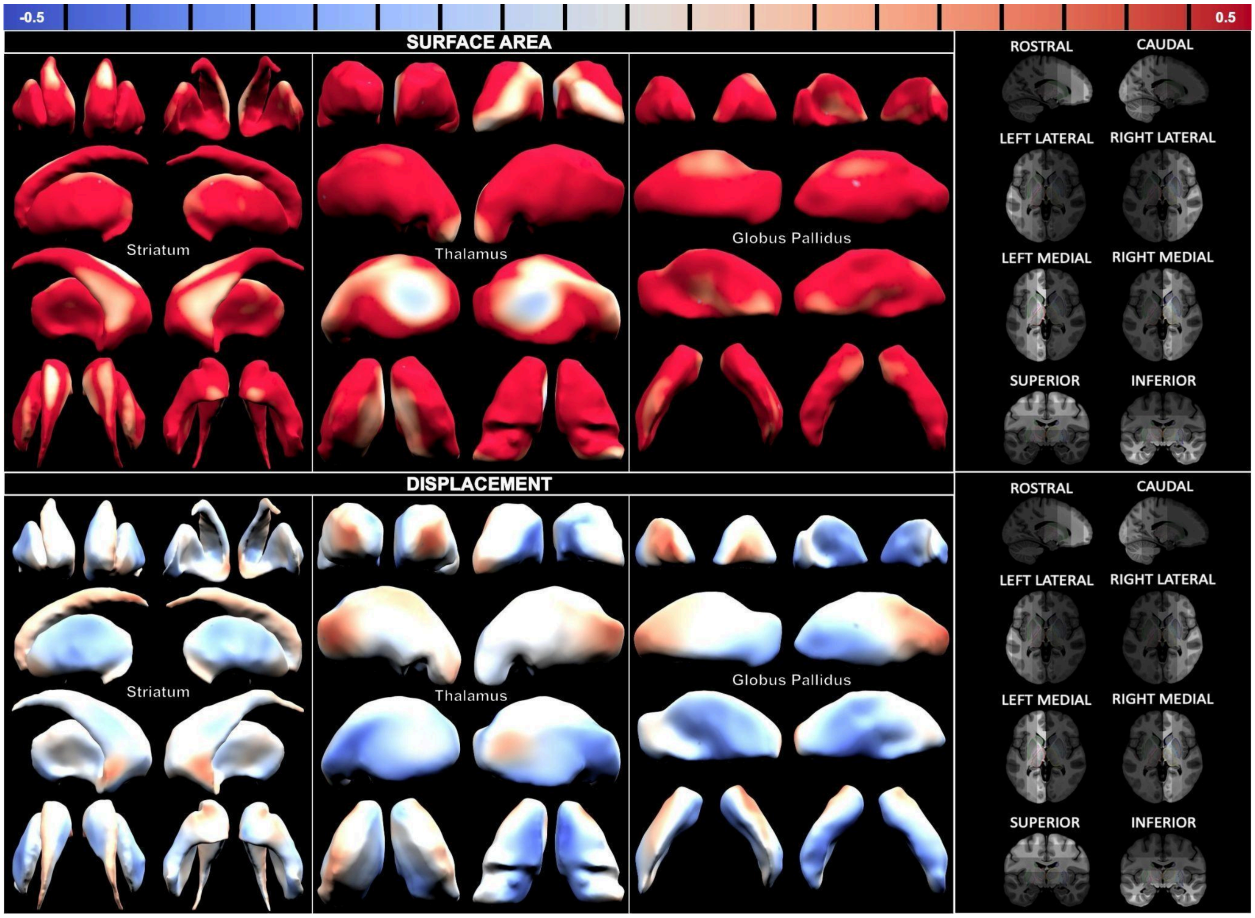

Figure S8. Genetic correlation (rg) between TBV and sex and age-adjusted vertex-wise surface area (top row) and displacement (bottom row) in the striatum, thalamus and globus pallidus. The views of the structures on display are shown in the column on the right-hand side. Ranges go from -0.5 (blue) to 0.5 (red).

Supplementary Tables

Table S1. Sample size, age, sex, average handedness, average fluid intelligence, average total brain volume and average ipsilateral subcortical structure specific volume for raw data (top-left), data after preprocessing and QC for brainmask and motion (top-right), data used for striatal analysis (center-left), data used for thalamic analysis (center-right) and data used for pallidal analysis (bottom-left).

	Preprocessed; QC for brain mask and motion (N=1033)				Subjects kept for striatal analysis (N = 821)			
	MZ	DZ	Non-Twin	Total	MZ	DZ	Non-Twin	Total
N	315	212	506	1033	232	168	421	821
Average age in years (±SD)	29.50 (3.34)	29.05 (3.50)	28.20 (3.87)	28.77 (3.69)	29.41 (3.35)	29.05 (3.40)	28.59 (3.83)	28.91 (3.63)
Age range (min-max)	22-36	22-36	22-37	22-37	22-36	22-35	22-37	22-37
Sex female:male	192:123	126:86	242:264	560:473	143:89	104:64	211:210	458:363
Average handedness (±SD)	79.52 (20.51)	79.04 (18.49)	77.14 (20.67)	78.26 (20.20)	78.67 (20.26)	79.70 (18.01)	77.69 (20.67)	78.38 (20.02)
Average fluid intelligence (±SD)	16.92 (4.57)	16.58 (5.05)	17.08 (4.81)	16.93 (4.79)	17.00 (4.57)	17.12 (4.75)	16.94 (4.85)	17.00 (4.75)
Average TBV (±SD)	1381909 (136408)	1410309 (155752)	1422232 (155333)	1407489 (150785)	1381677 (136553)	1407359 (146876)	1414289 (155168)	1403655 (148917)
Average left striatal volume (±SD)	9874 (969)	10154 (1111)	10204 (1036)	10093 (1042)	9871 (990)	10167 (1052)	10204 (1035)	10102 (1035)
Average right striatal volume (±SD)	10148 (990)	10407 (1119)	10472 (1061)	10360 (1061)	10142 (1011)	10410 (1048)	10469 (1053)	10365 (1049)
Average left thalamic volume (±SD)	6358 (552)	6454 (612)	6464 (613)	6430 (596)	6347 (555)	6452 (587)	6451 (600)	6422 (586)
Average right thalamic volume (±SD)	6319 (544)	6411 (608)	6407 (610)	6381 (591)	6302 (547)	6404 (584)	6401 (593)	6374 (580)
Average left pallidal volume (±SD)	1486 (142)	1535 (156)	1550 (159)	1527 (156)	1481 (145)	1534 (150)	1546 (158)	1525 (155)
Average right pallidal volume (±SD)	1369 (137)	1411 (144)	1429 (149)	1407 (147)	1364 (141)	1411 (140)	1427 (147)	1406 (146)

	Subjects kept for thalamic analysis (N = 866)				Subjects kept for pallidal analysis (N = 950)			
	MZ	DZ	Non-Twin	Total	MZ	DZ	Non-Twin	Total
N	249	174	443	866	268	193	489	950
Average age in years (±SD)	29.24 (3.35)	28.99 (3.43)	28.39 (3.86)	28.76 (3.65)	29.35 (3.37)	28.98 (3.46)	28.43 (3.83)	28.80 (3.65)
Age range (min-max)	22-36	22-35	22-36	22-36	22-36	22-35	22-37	22-37
Sex female:male	148:101	108:66	222:221	478:388	160:108	117:76	240:249	517:433
Average handedness (±SD)	79.59 (19.94)	79.39 (18.41)	77.21 (20.45)	78.35 (19.92)	79.82 (19.86)	79.66 (18.18)	77.59 (20.48)	78.65 (19.86)
Average fluid intelligence (±SD)	17.15 (4.42)	16.86 (4.88)	17.13 (4.79)	17.08 (4.70)	17.09 (4.50)	16.86 (4.96)	17.07 (4.80)	17.04 (4.75)

Average TBV (±SD)	1388202 (132675)	1410256 (147194)	1413508 (154164)	1405578 (147132)	1386315 (136804)	1415281 (151016)	1418705 (154990)	1408872 (149780)
Average left striatal volume (±SD)	9881 (946)	10146 (1094)	10159 (1007)	10076 (1015)	9892 (982)	10180 (1103)	10185 (1034)	10101 (1041)
Average right striatal volume (±SD)	10152 (962)	10400 (1107)	10430 (1038)	10344 (1037)	10165 (1002)	10430 (1114)	10454 (1062)	10368 (1063)
Average left thalamic volume (±SD)	6385 (541)	6459 (583)	6446 (597)	6431 (579)	6373 (555)	6480 (606)	6457 (608)	6438 (594)
Average right thalamic volume (±SD)	6342 (529)	6414 (587)	6393 (595)	6383 (575)	6331 (546)	6432 (606)	6405 (604)	6390 (589)
Average left pallidal volume (±SD)	1485 (139)	1533 (155)	1542 (155)	1524 (152)	1485 (144)	1538 (156)	1546 (158)	1527 (156)
Average right pallidal volume (±SD)	1369 (136)	1409 (146)	1423 (145)	1405 (144)	1368 (140)	1414 (144)	1426 (147)	1407 (146)

Table S2. Sample size, age, sex, average handedness, average fluid intelligence, average total brain volume and average ipsilateral subcortical structure specific volume for data used for PLSC analysis.

	Subjects kept for PLSC analysis			
	MZ	DZ	Non-Twin	Total
N	225	159	417	801
Average age in years (±SD)	29.48 (3.37)	29.20 (3.51)	28.47 (3.84)	28.90 (3.67)
Age range (min-max)	22-36	22-36	22-36	22-36
Sex female:male	138:87	104:55:00	221:196	463:338
Average handedness (±SD)	78.07 (21.03)	78.99 (19.16)	77.56 (21.03)	77.98 (20.65)
Average fluid intelligence (±SD)	17.29 (4.43)	16.56 (4.90)	16.70 (4.84)	16.84 (4.74)
Average TBV (±SD)	1375985 (133101)	1387553 (149628)	1399197 (152658)	1390365 (146973)
Average left striatal volume (±SD)	9840 (938)	10060 (1062)	10106 (1018)	10022 (1010)
Average right striatal volume (±SD)	10110 (951)	10323 (1079)	10371 (1042)	10288 (1030)
Average left thalamic volume (±SD)	6337 (556)	6371 (563)	6403 (600)	6378 (581)
Average right thalamic volume (±SD)	6296 (548)	6341 (568)	6345 (593)	6330 (575)
Average left pallidal volume (±SD)	1479 (140)	1523 (152)	1539 (160)	1519 (155)
Average right pallidal volume (±SD)	1363 (136)	1400 (144)	1419 (150)	1400 (147)

Table S3. Mean, standard deviation, minimal value, maximal value and number of unavailable (na) entries for each of the HCP variables used in the behavioral matrix of the PLSC analysis, across the HCP subjects included (N=801).

	Mean	Standard deviation	Min	Max	Number of NAs
Years of Education	14.96	1.79	11	17	2
Picture Sequence Memory	111.89	13.09	76.42	135.55	1
Card Sort	114.84	10.55	81.27	143.94	2
Flanker Inhibitory Control	111.45	10.08	84.9	142.11	0
PMAT Correct Responses (CR)	16.84	4.74	4	24	7
PMAT Skipped Items	3.08	3.85	0	15	7
PMAT Median Reaction Time (s)	15658.74	8980.46	2255	52155	7
Oral Reading Recognition	116.97	10.22	86.01	150.71	0
Picture Vocabulary	116.95	9.52	90.69	150.91	0
Pattern Comparison Processing Speed	114.68	15.33	51.62	154.69	0
AUC for Discounting of $200	0.26	0.2	0.02	0.98	7
AUC for Discounting of $40 K	0.51	0.28	0.02	0.98	7
SCPT: Median Response Time for True Positives	469.96	44.91	360	660	9
SCPT: Sensitivity	0.96	0.07	0	1	7
SCPT: Specificity	0.95	0.04	0.77	1	7
SCPT: Longest Run of Non-Responses	9.34	9.65	5	180	7
Penn Word Memory Test: CR	35.71	2.97	22	40	7
Penn Word Memory Test: Median Reaction Time for CRs (s)	1553.94	292.9	987.5	3192.5	7
9-hole Pegboard Dexterity	113.15	10.74	86.72	148.67	0
Anger Facial Recognition	6.78	1.02	2	8	7
Fear Facial Recognition	6.86	1.21	0	8	7
Happiness Facial Recognition	7.96	0.22	5	8	7
Neutral Facial Recognition	7.1	1.29	0	8	7
Sad Facial Recognition	6.79	1.16	2	8	7
Fear-Somatic Arousal	51.86	8.34	40.1	87.5	1
Anger-Affect	48.13	8.15	28.6	85.4	1
Anger-Hostility	50.56	8.51	36.6	74	1
Anger-Physical Aggression	51.93	8.84	43.4	83.1	1
Fear-Affect	50.52	8.06	32.9	84.9	1
Sadness	46.63	7.98	34.2	78.1	1
