# Supplementary material for: Morphological and anatomical variations in subcortical anatomy between humans and chimpanzees associated with heritability patterns related to human behavioral traits
Source: Commun Biol. 2026 Apr 21;9:852. doi: 10.1038/s42003-026-10066-6 (PMC13284390; doi:10.1038/s42003-026-10066-6)
Supplement: Supplementary file 2 — Reporting Summary [file 42003_2026_10066_MOESM2_ESM.pdf]

nature portfolio

Corresponding author(s): Nadia Blostein

Last updated by author(s): Apr 3, 2026

Reporting Summary

Nature Portfolio wishes to improve the reproducibility of the work that we publish. This form provides structure for consistency and transparency in reporting. For further information on Nature Portfolio policies, see our Editorial Policies and the Editorial Policy Checklist.

Statistics

For all statistical analyses, confirm that the following items are present in the figure legend, table legend, main text, or Methods section.

n/a	Confirmed	
☐	☒	The exact sample size (*n*) for each experimental group/condition, given as a discrete number and unit of measurement
☐	☒	A statement on whether measurements were taken from distinct samples or whether the same sample was measured repeatedly
☐	☒	The statistical test(s) used AND whether they are one- or two-sided<br>*Only common tests should be described solely by name; describe more complex techniques in the Methods section.*
☐	☒	A description of all covariates tested
☐	☒	A description of any assumptions or corrections, such as tests of normality and adjustment for multiple comparisons
☐	☒	A full description of the statistical parameters including central tendency (e.g. means) or other basic estimates (e.g. regression coefficient) AND variation (e.g. standard deviation) or associated estimates of uncertainty (e.g. confidence intervals)
☐	☒	For null hypothesis testing, the test statistic (e.g. F, t, r) with confidence intervals, effect sizes, degrees of freedom and P value noted<br>*Give P values as exact values whenever suitable.*
☐	☒	For Bayesian analysis, information on the choice of priors and Markov chain Monte Carlo settings
☐	☒	For hierarchical and complex designs, identification of the appropriate level for tests and full reporting of outcomes
☐	☒	Estimates of effect sizes (e.g. Cohen's *d*, Pearson's *r*), indicating how they were calculated

Our web collection on statistics for biologists contains articles on many of the points above.

Software and code

Policy information about availability of computer code

Data collection	Human MRI and behavioral data were obtained from the Human Connectome Project (HCP) S1200 release, and chimpanzee MRI data were obtained from the National Chimpanzee Brain Resource (NCBR), as described in the Methods.
Data analysis	Data were processed and analyzed using a combination of open-source and custom software. Image preprocessing and segmentation were performed using MAGeT Brain, ANTs, and minc-bpipe. Heritability analyses were conducted using OpenMx in R, and partial least squares correlation analyses were performed using pyls in Python. Custom scripts used for subcortical surface mapping and statistical analyses are available from the authors upon reasonable request.

For manuscripts utilizing custom algorithms or software that are central to the research but not yet described in published literature, software must be made available to editors and reviewers. We strongly encourage code deposition in a community repository (e.g. GitHub). See the Nature Portfolio guidelines for submitting code & software for further information.

Data

Policy information about availability of data

All manuscripts must include a data availability statement. This statement should provide the following information, where applicable:

- Accession codes, unique identifiers, or web links for publicly available datasets
- A description of any restrictions on data availability
- For clinical datasets or third party data, please ensure that the statement adheres to our policy

Human data are available from the Human Connectome Project (HCP) S1200 release, and chimpanzee MRI data are available from the National Chimpanzee Brain Resource (NCBR), subject to their respective data use agreements. Derived data supporting the findings of this study are available from the authors upon reasonable request.

Research involving human participants, their data, or biological material

Policy information about studies with human participants or human data. See also policy information about sex, gender (identity/presentation), and sexual orientation and race, ethnicity and racism.

Reporting on sex and gender	The HCP sample includes both male and female participants; sex is used as a covariate in statistical models. Analyses are not stratified by sex; instead, sex effects are adjusted for in heritability and vertex‐wise models.
Reporting on race, ethnicity, or other socially relevant groupings	No race/ethnicity variables are analysed or reported in the article; groupings by race/ethnicity are not used.
Population characteristics	Participants: 1,113 healthy young adults from the HCP S1200 release.<br>Age: young adult range (HCP typical range is ~22–35 years; article describes them as “healthy young adults”).<br>All participants are free of major neurological or psychiatric disorders, as per HCP inclusion criteria.
Recruitment	Recruitment procedures, inclusion/exclusion criteria, and consent procedures were handled by the Human Connectome Project consortium; participants volunteered for HCP scanning and behavioural testing under HCP protocols.
Ethics oversight	HCP studies were approved by the Washington University institutional review board and associated ethics boards; all participants gave informed consent (as per HCP documentation). The present analyses were conducted under HCP’s data‐use agreements. and under local ethics approvals at the authors’ institutions for analysis of de‐identified HCP data.

Note that full information on the approval of the study protocol must also be provided in the manuscript.

Field-specific reporting

Please select the one below that is the best fit for your research. If you are not sure, read the appropriate sections before making your selection.

☐ Life sciences ☐ Behavioural & social sciences ☒ Ecological, evolutionary & environmental sciences

For a reference copy of the document with all sections, see nature.com/documents/nr-reporting-summary-flat.pdf

Ecological, evolutionary & environmental sciences study design

All studies must disclose on these points even when the disclosure is negative.

Study description	Quantitative, observational neuroimaging study relating subcortical structure (volumes and vertex‐wise morphometry) to genetics (heritability) and a large battery of cognitive, affective and motor behavioural measures in healthy young adults.
Research sample	HCP healthy young adults (1,113 participants) with high‐quality structural MRI and behavioural testing.<br>Demographics (age range, sex distribution) are described in the main text or Methods of the article.
Sampling strategy	Convenience sample defined by the HCP S1200 release; no additional sampling by the authors.<br>Sample size determined by the available HCP dataset; the large cohort is sufficient for estimating heritability and multivariate brain–behaviour relationships.
Data collection	MRI data: high‐resolution structural MRI acquired at HCP imaging centres using standardized protocols.<br>Behavioural data: 30 behavioural attributes from HCP (cognitive, emotional, motor instruments) including fluid intelligence, language tasks, negative affect, facial recognition, etc.
Timing and spatial scale	Data collection dates and detailed timelines are as per HCP acquisition protocols; the present study uses existing datasets and does not collect new data. Spatial scale dependant on reosolution of MRI data.

Data exclusions | Any participants failing HCP QC or subcortical segmentation/surface reconstruction were excluded; final N = 1,113.

Reproducibility | This is a single, large cohort study; results (heritability maps, PLSC patterns, interspecies differences) are internally supported by multiple converging analyses rather than an independent replication cohort.

Randomization | There are no experimental groups; all participants belong to a single observational cohort, so randomization is not applicable.

Blinding | MRI and behavioural data were acquired by HCP with standardized procedures. Analyses were fully automated (segmentation, surface estimation, statistical modelling) and do not involve subjective rating requiring blinding.

Did the study involve field work? ☐ Yes ☒ No

Reporting for specific materials, systems and methods

We require information from authors about some types of materials, experimental systems and methods used in many studies. Here, indicate whether each material, system or method listed is relevant to your study. If you are not sure if a list item applies to your research, read the appropriate section before selecting a response.

Materials & experimental systems

n/a	Involved in the study	
☒	☐	Antibodies
☒	☐	Eukaryotic cell lines
☒	☐	Palaeontology and archaeology
☒	☐	Animals and other organisms
☒	☐	Clinical data
☒	☐	Dual use research of concern
☒	☐	Plants

Methods

n/a	Involved in the study	
☒	☐	ChIP-seq
☒	☐	Flow cytometry
☐	☒	MRI-based neuroimaging

Plants

Seed stocks | not applicable

Novel plant genotypes | not applicable

Authentication | not applicable

Magnetic resonance imaging

Experimental design

Design type | MRI

Design specifications | See methods

Behavioral performance measures | See Methods

Acquisition

Imaging type(s) | T1w

Field strength | 3T

Sequence & imaging parameters | TE/TR=2.14/2400 ms, TI=1000ms, a=8o, 0.7 mm3 isotropic voxels, HCP S1200 Reference Manual

Area of acquisition | HCP S1200 Reference Manual

Diffusion MRI ☐ Used ☐ Not used

Preprocessing

Preprocessing software	HCP structural pipelines for initial preprocessing (brain extraction, bias correction, alignment).<br>MAGeT Brain and associated tools for subcortical segmentation and surface generation.
Normalization	Data registered to a human template (for humans) and a chimpanzee template using linear and nonlinear registrations.<br>A human–chimpanzee hybrid template was constructed to derive deformation fields for mapping human subcortical surfaces into chimpanzee space.
Normalization template	Human: HCP template / MAGeT brain atlas space; chimpanzee: chimpanzee MRI template; hybrid template for interspecies mapping.
Noise and artifact removal	Standard HCP structural preprocessing for artifact and noise; additional QC steps before segmentation and surface‑based analyses.
Volume censoring	Not applicable; structural MRI only.

Statistical modeling & inference

Model type and settings	Mass‑univariate vertex‑wise heritability maps.
Effect(s) tested	Multivariate PLSC relating vertex‑wise measures to behavioural variables.

Specify type of analysis: ☐ Whole brain ☐ ROI-based ☒ Both

Anatomical location(s)	Striatum, thalamus, globus pallidus
Statistic type for inference (See Eklund et al. 2016)	Voxel-wise surface area and displacmeent
Correction	Vertex‑wise maps thresholded using FDR 5% for significance. Cross‑analysis correlations corrected with FDR 5% across 42 comparisons using the subcortical spin test.

Models & analysis

n/a	Involved in the study
☒	☐ Functional and/or effective connectivity
☒	☐ Graph analysis
☐	☒ Multivariate modeling or predictive analysis

Multivariate modeling and predictive analysis	We performed a Partial Least Squares Correlation (PLSC) analysis on the HCP subjects to generate latent variables (LV) relating vertex-wise surface area and displacement in the subcortical structures of interest to behavior. The PLSC algorithm applies a singular value decomposition to the covariance matrix between two sets of Z-scored input features: the brain features (vertex-wise surface area and displacement, examined separately in this study and residualized for sex and age) and behavioral features (30 attributes selected from the HCP restricted behavioral and demographic data, residualized for sex and age). Pyls 0.01 (https://github.com/rmarkello/pyls) was used to run the PLSC analysis in Python 3.6.8. Each LV captures what linear combination of brain shape measurements covaries the most with what linear combination of behavioral attributes.
